# Massive MIMO NOMA: Double-Mode Model towards Green 5G Networks

**DOI:** 10.3390/s23146425

**Published:** 2023-07-15

**Authors:** Preksha Jain, Akhil Gupta, Sudeep Tanwar, Fayez Alqahtani, Maria Simona Raboaca, Wael Said

**Affiliations:** 1 School of Electronics and Electrical Engineering, Lovely Professional University, Phagwara 144411, India; prekshajain248@gmail.com; 2 Department of Computer Science and Engineering, Institute of Technology, Nirma University, Ahmedabad 382481, India; 3 Software Engineering Department, College of Computer and Information Sciences, King Saud University, Riyadh 12372, Saudi Arabia; fhalqahtani@ksu.edu.sa; 4 Doctoral School, University Politehnica of Bucharest, Splaiul Independentei Street, No. 313, 060042 Bucharest, Romania; simona.raboaca@icsi.ro; 5 National Research and Development Institute for Cryogenic and Isotopic Technologies—ICSI Rm. Vâlcea, Uzinei Street, No. 4, 240050 Râmnicu Vâlcea, Romania; 6 Computer Science Department, Faculty of Computers and Informatics, Zagazig University, Zagazig 44511, Egypt; wael.mohamed@zu.edu.eg

**Keywords:** 5G green network, energy efficiency, mMIMO NOMA

## Abstract

With the development of the Internet of Things (IoT), the number of devices will also increase tremendously. However, we need more wireless communication resources. It has been shown in the literature that non-orthogonal multiple access (NOMA) offers high multiplexing gains due to the simultaneous transfer of signals, and massive multiple-input–multiple-outputs (mMIMOs) offer high spectrum efficiency due to the high antenna gain and high multiplexing gains. Therefore, a downlink mMIMO NOMA cooperative system is considered in this paper. The users at the cell edge in 5G cellular system generally suffer from poor signal quality as they are far away from the BS and expend high battery power to decode the signals superimposed through NOMA. Thus, this paper uses a cooperative relay system and proposes the mMIMO NOMA double-mode model to reduce battery expenditure and increase the cell edge user’s energy efficiency and sum rate. In the mMIMO NOMA double-mode model, two modes of operation are defined. Depending on the relay’s battery level, these modes are chosen to utilize the system’s energy efficiency. Comprehensive numerical results show the improvement in the proposed system’s average sum rate and average energy efficiency compared with a conventional system. In a cooperative NOMA system, the base station (BS) transmits a signal to a relay, and the relay forwards the signal to a cluster of users. This cluster formation depends on the user positions and geographical restrictions concerning the relay equipment. Therefore, it is vital to form user clusters for efficient and simultaneous transmission. This paper also presents a novel method for efficient cluster formation.

## 1. Introduction

The mobile communication system trends show a high rise in mobile communications devices, network services, and data transfer volume. This trend is anticipated to further increase, with an increase in new services incorporating machine-to-machine (M2M) types of communication. Additionally, with the development of cloud-based services, advanced multimedia applications (augmented reality, virtual reality, etc.), and Internet of Things (IoT) services, data traffic is predicted to increase [1] dramatically [2]. As a result, the 5G green networks need to achieve a 10-to-100-fold rise in data rates and device connections, and up to 99.99% network availability compared with the previous wireless communication generations. Such intensified requirements will tremendously increase the energy consumption of the system. Therefore, the 5G green networks need to realize the high requirements of data rates while saving system energy [3,4].

The current scenario requires a 10-fold reduction in energy consumption in the 5G green networks compared to previous generations, with the efficient incorporation of novel 5G techniques with the prevailing wireless technologies [5,6,7]. Therefore, energy concerns are the primary concern when realizing a 5G green network.

The mMIMO and non-orthogonal multiple access (NOMA) are the key technologies of 5G networks. The MMO exploits multi-path propagation by employing multiple antennas, which can help to increase the system’s capacity, security, spectrum efficiency, and reliability. By combining multiple users in a single resource block, NOMA aids in improving the spectrum efficiency, capacity, latency, and fairness of the system [8,9,10]. A single-input–single-output (SISO) system employs a single antenna at both the base station (BS) and at all the user equipment (UEs). At the same time, multiple-input–multiple-outputs (MIMOs) use multiple antennas to offer a higher reliability and capacity in terms of multiplexing, array, and diversity gains [11]. The massive-multiple-input–multiple-outputs (mMIMO) use a much larger number of antennas than conventional MIMO systems. Diversity gains are increased at the mMIMO scale with several independent channels between BS and UEs, while the maximum multiplexing gain is decreases with the number of antennas at BS and UEs.

In NOMA, multiple users are grouped in a single cluster, and the signals of all the users are superimposed to form a single signal, which is transmitted to all the users in the cluster. In this way, the signals of multiple users are transmitted simultaneously, which saves spectrum resources and increases the system’s capacity. The transmitter takes care of fair resource allocation by assigning powers depending on the channel gains of the UEs. The UE receives its signal through successive interference cancellation (SIC), where the strongest signal is decoded first and then deducted from the superimposed signal. A UE repeats this process multiple times to obtain the intended signal from the superimposed signal, and the weaker signals are considered interference.

The advantage of NOMA over the system is that it can achieve massive connectivity and reduce the cost of signaling and simultaneous transmission to a group of users. Additionally, the UEs in NOMA need not make scheduling requests to the BS, reducing latency [10]. Moreover, NOMA can work with the present techniques in wireless communication systems, attracting strong research interest in this field.

An explosion in the communication systems’ mobile traffic, triggered by new network service demands, has swiftly activated increases in energy consumption, meaning that energy-saving is a vital part of the cellular communication system design. This paper exploits the cooperative relay technique for system design. The cooperative relay technique assists the transmitted signal in reaching the receiver through the forwarding technique. For example, in a cellular system, if the signal transmitted by the base station (BS) is unable to reach the users located at the cell edge, then another user acts as a relay (which is typically situated between the BS and the user at the cell-edge) to forward the signal from the BS to the user at the cell edge. Such a system is lucrative as the power used to transmit the signal from the BS is now rescued, as the signal has to travel a shorter distance to the relay. This helps to efficiently utilize the system’s energy. Moreover, the corporative relay system aids in providing a wider coverage area and enhancing link reliability [12].

**Figure 1:**
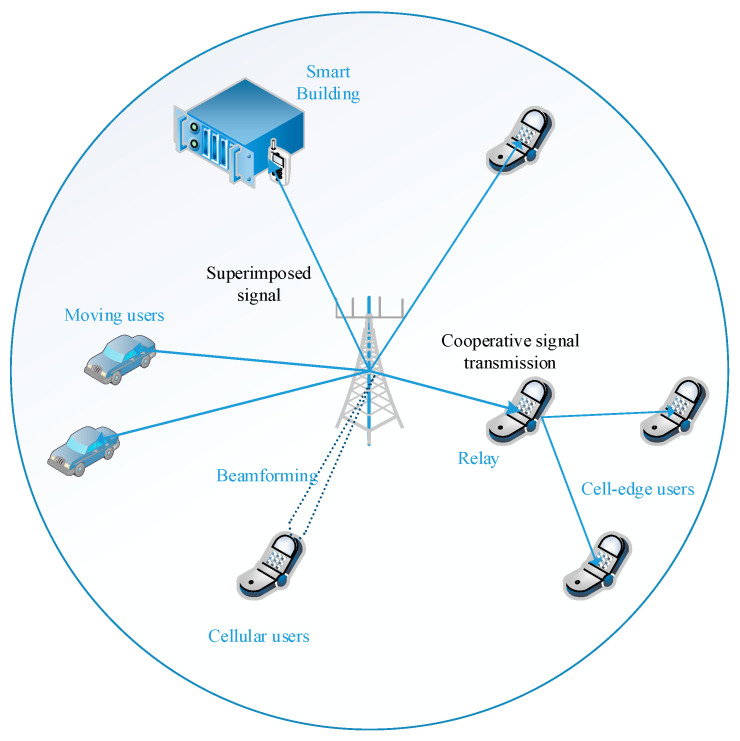
A conventional mMIMO NOMA system.

This paper combines mMIMO, NOMA, and cooperative relay techniques to extract their full advantage and proposes an mMIMO NOMA double-mode model. This proposed model works in two modes, which are chosen according to the battery power of the relay, to efficiently utilize the energy of the system. Both the relays and the end-users both have limited handset batteries. Therefore, the proposed system enhances the energy efficiency of the system by saving the battery power of the UEs. The simulation results show that the proposed system can improve the sum rate and energy efficiency and reduce the battery power consumption of the cell-edge user. A conventional mMIMO NOMA system is shown in Figure 1.

### 1.1. Research Background

Lately, little research has been carried out to validate the superiority of NOMA performance compared to conventional schemes. The traditional orthogonal multiple access (OMA) schemes cannot satisfy the demands of 5G systems’ spectral efficiency, rates, and capacity. The literature studies on the NOMA system, MIMO NOMA system, and mMIMO NOMA systems shows ameliorations in energy efficiency, resource allocation, rates and capacity, compared to traditional systems.

The concept of NOMA was first presented in [13], where the authors show that the spectral efficiency and fairness of NOMA systems are far better than those of OMA systems. In [14], a comparison analysis is carried out of OMA and NOMA systems regarding fairness and sum rate. In contrast, the authors in [15] compare the average sum capacity of OMA and NOMA power line communication systems. Power allocation in NOMA is studied in [16] to enhance global energy efficiency. A dynamic resource allocation strategy is investigated in [17] to optimize the energy efficiency of the NOMA system. A novel technique of multiple interference cancellation (MIC) is proposed in [18] to optimize energy and reduce complexity in device-to-device (D2D)-NOMA systems. A two-user MIMO NOMA system is considered in [19], and the numerical analysis proves that the sum rates achieved by the MIMO NOMA system are superior to those achieved by MIMO OMA systems.

In contrast, the authors in [18] compare fairness in two-user MIMO OMA and MIMO NOMA systems through a novel power allocation strategy. A mimo NOMA system [20,21] carries out a capacity and rate analysis. To the best of our knowledge, an efficient clustering scheme and reduction in battery expenditure have yet to be considered. It is important to efficiently utilize the energy of the system and form clusters for system operation, considering the user’s locations and antenna parameters. In this paper, the proposed efficient clustering technique investigates the performance of the communication system by further proposing the mMIMO NOMA double-mode model based on relay power. The proposed scheme has potential applications in broadcasting, multicasting, cellular data transfer, etc. Table 1 shows a comparative analysis of the proposed and existing techniques in the literature.

### 1.2. Contributions

According to the literature survey, a practical scenario of analyzing NOMA or mMIMO NOMA systems, considering the battery power of the UEs, needs to be presented. Therefore, to demonstrate a comparative assessment of mMIMO NOMA with the proposed mMIMO NOMA double-mode model, practical considerations of battery equipment power are considered in this paper. The key contributions are as follows:

- The relay and cell-edge user locations bound the user clustering in the NOMA system. Hence, it is important to efficiently form clusters for system operation, depending on the user’s location. A novel clustering technique is proposed in this paper, which exploits the antenna parameters of the relay device to group cell-edge users into a cluster.
- A mMIMO NOMA double-mode model is proposed to reduce the battery expenditure and increase the cell edge user’s energy efficiency and the sum rate. In the mMIMO NOMA double-mode model, two modes of operation are defined, and these modes are chosen depending on the battery level of the relay to utilize the energy of the system efficiently. We compared the mMIMO NOMA double-mode model for static user and mobile user cases.

The simulation shows that the proposed mMIMO NOMA double-mode model is superior to the mMIMO NOMA technique in terms of sum rate, cell-edge user battery consumption, and energy efficiency.

### 1.3. Organization and Notations

The remaining paper is organized as follows. Section 2 describes the system model. The proposed method mMIMO NOMA double-mode model is expanded in Section 3. Section 4 validates the simulation results and the proposed techniques’ efficiency. The paper finally concludes in Section 5. The numerous notations used in the document are provided in Table 2.

## 2. System Model

**Figure 2:**
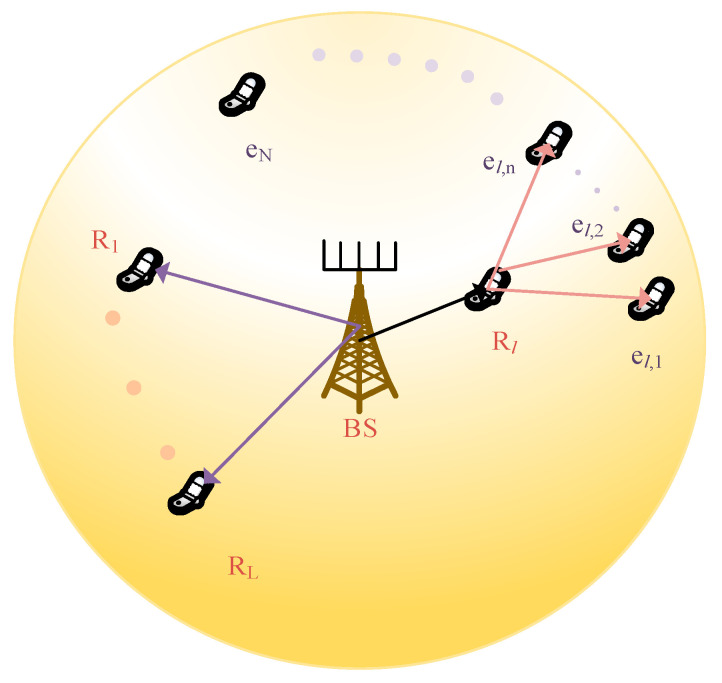
System description.

We consider a scenario of a single cell downlink mMIMO NOMA network, as depicted in Figure 2. It is supposed that the users are arbitrarily disseminated in the cell, with the total number of users denoted by Ň. The total number of antennas at the BS is denoted by Ä. The total number of single antenna relays and cell-edge users is denoted by L and N respectively. The set of L relay users is denoted as Ψ = {$R_{1},R_{2},\ldots ,R_{L}$}. The battery power of $R_{l}$, where $R_{l}\in \Psi$ is denoted as $P_{l}$. The mMIMO NOMA uses superposition coding at the transmitter and is employed at the receiver SIC. Each cluster contains a maximum of n users. The $R_{l}$ serves a maximum of n cell-edge users at a time. The BS superimposes the signals, and ${Ɓ}_{max}$ is the maximum power of BS. The set of N cell-edge users is represented as ƣ = {$e_{1},e_{2},\ldots ,e_{N}$}. The cell-edge users are grouped in clusters using the angle selection scheme (ASS) [22,23,24,25,26,27] and a total of L clusters are assumed. All the users are assumed to be moving with a velocity v m/s. The system is observed in a time frame of T sec. At maximum n, cell-edge users are grouped, and served by $R_{l}$, as denoted in set ${ƣ}_{l}$ = {$e_{l,1},e_{l,2},\ldots ,e_{l,n}$}. It takes two-time slots to complete communication BS to the cell-edge-user. The BS sends the superimposed signal $x_{l}$ to the $R_{l}$ in the first time slot, and in the second time slot, the $R_{l}$ forwards it to the cluster of the cell-edge users. It is assumed that the BS has complete channel state information (CSI) and the considered scenario is a quasi-static Rayleigh fading. The signal is transmitted by the BS to $R_{l}$, with, power $a_{l}Ɓ$, where $\sum_{n=1}^{N}a_{l,n}Ɓ<{Ɓ}_{\max}$. Unlike NOMA, the mMIMO NOMA system consists of two steps.

In the first step, the superimposed signal $x_{l}$ is constructed and transmitted to $R_{l}$; this is given as

(1) $$x_{l}\left(t\right)=\sum_{n=1}^{N}\sqrt{a_{l,n}Ɓ}\;x_{l,n}\left(t\right)$$

where $x_{l,n}\left(t\right)$ depicts the signal of $e_{l,n}$ at time instance t. Here, $a_{l,n}$ is the power allocation coefficient.

We usd zero-forcing beamforming at the BS. To balance implementation complexity with the system performance, we designed a weight $w_{l}$ $\in {\mathbb{C}}^{Ä\mathrm{xL}}$ for lth cluster. In the second step, the BS constructs the total transmit signal $x_{BS}$ of all the relays as follows:

(2) $$x_{BS}\left(t\right)=\sum_{l=1}^{L}w_{l}x_{l}\left(t\right)$$

where $w_{l}$ denotes the transmit beam design for $R_{l}$.

Then, the BS broadcasts the $x_{BS}$ over downlink channels. Consecutively, the signal received in the first time slot, at $R_{l}$, is given as

(3) $$y_{R_{l}}\left(t\right)=h_{l}^{H}\left(t\right)w_{l}\left(t\right)x_{l}\left(t\right)+\sum_{j=1,j\neq l}^{L}h_{l}^{H}\left(t\right)w_{j}\left(t\right)x_{j}\left(t\right)+n_{R_{l}}$$

where the matrix $h_{l}\in {\mathbb{C}}^{Ä\mathrm{xL}}$ represents the channel between BS and $R_{l}$, $h_{l}^{H}$ represents the Hermitian matrix of $h_{l}$ and $w_{l}$ represents the projection of $h_{l}$ at time instance t. For simplicity, we omit the use of notation t. The additive white Gaussian noise (AWGN) in the link between BS and relay is represented by $n_{l}$, with mean zero and $\sigma^{2}$ variance, such that $n_{l}$~ *CN* (0,$\sigma^{2}$). Since the beams are perfect, i.e., $h_{l}^{H}w_{l}=1$ and $h_{j}^{H}w_{j}=0$ ∀l≠j, from (1) and (3), the signal at the output can be expressed as

(4) $$y_{R_{l}}=h_{l}^{H}w_{l}\sqrt{a_{l,n}Ɓ}\;x_{l,n}+h_{l}^{H}w_{l}\overset{N}{\underset{i=n+1}{\sum }}\sqrt{a_{l,i}Ɓ}\;x_{l,i}\;+h_{l}^{H}w_{l}\sum_{k=1}^{n-1}\sqrt{a_{l,k}Ɓ}\;x_{l,k}+n_{R_{l}}$$

where $h_{l}^{H}w_{l}\sqrt{a_{l,n}Ɓ}\;x_{l,n}$, represents the desired signal of nth user in lth cluster, $h_{l}^{H}w_{l}\sum_{i=n+1}^{N}\sqrt{a_{l,i}Ɓ}\;x_{l,i}$ represents interference from other users, and $h_{l}^{H}w_{l}\sum_{k=1}^{n-1}\sqrt{a_{l,k}Ɓ}\;x_{l,k}$ represents interference due to an imperfect SIC. The term $h_{l}^{H}w_{l}\sum_{k=1}^{n-1}\sqrt{a_{l,k}Ɓ}\;x_{l,k}$ equals zero in the case of a perfect SIC.

In the second time slot, $R_{l}$ re-encodes the received signal and assigns power to these signals before forwarding. The set $b=\left\{b_{1},b_{2,}\ldots ,b_{n}\right\}$, expresses the power coefficient, assigned by $R_{l}$, for a group of n cell-edge users. The $b_{k}P_{l}$ denotes the power assigned by $R_{l}$ to the corresponding cell-edge users such that $\sum_{k=1}^{n}b_{k}P_{l}\leq P_{l_{max}}.$ $P_{l_{max}}$ signifies the maximum power of $R_{l}$.

The $R_{l}$ superimposes the signals and forwards the superimposed signal to the cluster of n cell-edge users, given as

(5) $${Ƒ}_{l}=\sqrt{b_{n}P_{l}}x_{l}$$

The received signal at $e_{l,n}$ is given as

(6) $$y_{e_{l,n}}=g_{n}\sqrt{b_{n}P_{l}}x_{l}+g_{n}\underset{\begin{array}{c} k\epsilon \Psi ,i\epsilon ƣ\\ k\neq n\\ g_{n}P_{l}<g_{i}P_{k} \end{array}}{\sum }\sqrt{b_{i}P_{k}}x_{i}\;+g_{n}\sum_{k=1}^{n-1}\sqrt{b_{k}P_{l}}x_{k}+n_{e_{l,n}}$$

where $g_{n}$ and $n_{e_{l,n}}$ signify the channel and relay-end-user AWGN, respectively. The $n_{e_{l,n}}$ is with zero mean and variance $\sigma_{e_{l,n}}{}^{2}$, such that $n_{e_{l,n}}$ ~ *CN* (0,$\sigma_{e_{l,n}}{}^{2}$) between $R_{l}$ and $e_{l,n}$. In Equation (6), $g_{n}\sqrt{b_{n}P_{l}}x_{l}$, represents the desired superimposed signal at $e_{l,n}$, $g_{n}\underset{\begin{array}{c} k\epsilon \Psi ,i\epsilon ƣ\\ k\neq n\\ g_{n}P_{l}<g_{i}P_{k} \end{array}}{\sum }\sqrt{b_{i}P_{k}}x_{i}$ represents interference from other users, and $g_{n}\sum_{k=1}^{n-1}\sqrt{b_{k}P_{l}}x_{k}$ represents interference due to an imperfect SIC, which equals zero in the case of a perfect SIC.

### 2.1. Sum Rate

The rates are evaluated with the achieved SINR of the signal. The SINR achieved at $R_{l}$ is given as

(7) $$SINR_{\;R_{l}\;}=\frac{Ɓa_{l,n}{\left|h_{l}^{H}w_{l}\right|}^{2}}{\sum_{i=n+1}^{N}Ɓa_{l,i}{\left|h_{l}^{H}w_{l}\right|}^{2}+\sum_{k=1}^{n-1}a_{l,k}Ɓ{\left|h_{l}^{H}w_{l}\right|}^{2}+\sigma^{2}}$$

The SINR achieved at $e_{l,n}$ is given as

(8) $$SINR_{e_{l,n}\;}=\frac{b_{n}P_{l}{\left|g_{n}\right|}^{2}}{\sum_{\begin{array}{c} k\epsilon \Psi ,i\epsilon ƣ\\ l\neq k,i\neq n\\ g_{n}P_{l}<g_{i}P_{k} \end{array}}\sqrt{b_{i}P_{k}}{\left|g_{n}\right|}^{2}+\sum_{k=1}^{n-1}b_{k}P_{l}{\left|g_{n}\right|}^{2}+\sigma_{e_{l,n}}{}^{2}}$$

The end-to-end SINR of the mMIMO-NOMA relay system is given as the minimum SINRs of BS-$R_{l}$ and $R_{l}-e_{l,n}$ links, i.e.,

(9) $$SINR_{E2E\left(n\right)}=\min\left(SINR_{R_{l}},\;SINR_{e_{l,n}}\right)$$

The achieved data rate of the BS-$R_{l}$ and $R_{l}-e_{l,n}$ end-to-end link is given as

(10) $${Ð}_{E2E\left(n\right)}=log_{2}\left(1+SINR_{E2E\left(n\right)}\right)\;\mathrm{bps}/\mathrm{Hz}$$

Therefore, the achievable sum-rate is expressed as

(11) $${Ð}_{E2E}{}^{T}=\sum_{i=1}^{N}{Ð}_{E2E\left(i\right)}\;\mathrm{bps}/\mathrm{Hz}$$

### 2.2. Energy Efficiency

With the development of mobile communication systems, the number of devices and device-to-device connections also increases, which leads to an increase in data-traffic. This results in high power consumption in the devices. Therefore, it is important to reduce power consumption for the expansion of green communication systems. The total power utilized by $e_{l,n}$, is given as(12)
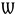
_*l*,*n*_ = Ϫ_*n*_*b_n_P_l_* + ҏ_*n*_
Here, amplifier drain efficiency at $e_{l,n}$ is denoted by ${Ϫ}_{n}$. The power spent at the circuit of $e_{l,n}$ is signified by ${ҏ}_{n}$, and is given as

(13) $${ҏ}_{n}={ҏ}_{n}^{det}+{ҏ}_{n}^{co}$$

where ${ҏ}_{n}^{det}$ and ${ҏ}_{n}^{co}$ represent power consumption at $e_{l,n}$ by its detector and decoder circuits, respectively.

For the development of green communication systems, the optimization of energy efficiency is crucial. Therefore, we analyze the energy efficiency of the system as the ratio of the system’s data rate to the power spent.

Energy consumption at $R_{l}$, utilized for processing and forwarding the signal for $e_{l,n}$, is signified by ${Ϸ}_{l}$ and is given as

(14) $${Ϸ}_{l}={Ԃ}_{l}(b_{n}P_{l})+{Ƥ}_{l}$$

Here, the amplifier drain efficiency is denoted by ${Ԃ}_{l}$, and ${Ƥ}_{l}$ represents the circuit power consumption, of the $R_{l}$, which is given as

(15) $${Ƥ}_{l}={Ƥ}_{l}^{det}+{Ƥ}_{l}^{co}+{Ƥ}_{l}^{reg}$$

where ${Ƥ}_{l}^{det},\;{Ƥ}_{l}^{co}$ and ${Ƥ}_{l}^{reg}$ are the detector, decoder, and regenerator circuit power consumption at $R_{l}$, respectively.

The energy efficiency for transmitting signals to $e_{l,n}$ is given as

(16) $$EE_{l,n}=\frac{{Ð}_{E2E\left(n\right)}}{{Ϸ}_{l}}$$

The energy efficiency for transmitting the signal to N cell-edge users is given as

(17) $$EE^{T}=\frac{{Ð}_{E2E}{}^{T}}{\sum_{i=1}^{N}{Ϸ}_{l}}$$

## 3. Massive MIMO Double-Mode Model

The mMIMO NOMA double-mode model chooses the modes of operation depending on the cooperative relay battery power.

The proposed mMIMO NOMA mMIMO NOMA double-mode model has two operational modes, denoted by ${ʩ}_{s},\;s=\left(1,2\right)$. The range of battery level of $R_{l}$ relay equipment is represented as $P_{l}^{{ʩ}_{s}}$ (working in the mode ${ʩ}_{s}$), such that $P_{l}^{{ʩ}_{1}}\leq \;P_{l}^{{ʩ}_{2}}$. Each relay places the cell-edge users in a cluster for signal transmission on the condition that the cell-edge user is located within the relay range and does not fall into the cluster formed by other relays. The two modes of operation are defined as follows:

${ʩ}_{1}$: when the power of $R_{l}$ is in the $P_{l}^{{ʩ}_{1}}$ range, then the operational mode ${ʩ}_{1}$ is chosen. The BS sends the superimposed signal $x_{l}$ to $R_{l}$ and $x_{BS}$ to all the relays for a cluster of n cell-edge users. Then, $R_{l}$ forwards the super-imposed signal ${Ƒ}_{l}$ given in Equation (5) to each user in the cluster n cell-edge users, acting as an amplify and forward relay. The superimposed signal ${ŷ}_{e_{l,n}}$ is acknowledged at each cell-edge user. Each cell-edge user obtains signal $y_{e_{l,n}}$ after SIC to extract $x_{l}$.

${ʩ}_{2}$: when the power of $R_{l}$ is in the range $P_{l}^{{ʩ}_{2}}$, then operational mode ${ʩ}_{2}$ is chosen. The BS sends the superimposed signal $x_{l}$ to $R_{l}$ and $x_{BS}$ to all the relays for a cluster of n cell-edge users. In this mode, the $R_{l}$ decodes the signal of each user in the cluster and forwards it to each user in the cluster, acting as a decode and forward relay. The signal transmitted $R_{l}$ in Equation (5) becomes ${Ƒ}_{l}=\sqrt{b_{n}P_{l}}x_{l,n}$. The signal received at $e_{l,n}$ is given as

(18) $$y_{e_{l,n}}=g_{n}\sqrt{b_{n}P_{l}}x_{l,n}+\sum_{\begin{array}{c} k\epsilon \Psi ,i\epsilon ƣ\\ l\neq k,i\neq n\\ g_{n}P_{l}<g_{i}P_{k} \end{array}}\sqrt{b_{i}P_{k}}+n_{e_{l,n}}$$

The SINR achieved at $e_{l,n}$ under perfect SIC is given as

(19) $$SINR_{e_{l,n}\;}=\frac{b_{n}P_{l}{\left|g_{n}\right|}^{2}}{\sum_{\begin{array}{c} k\epsilon \Psi ,i\epsilon Ɓ\\ l\neq k,i\neq n\\ g_{n}P_{l}<g_{i}P_{k} \end{array}}\sqrt{b_{i}P_{k}}{\left|g_{n}\right|}^{2}+\sigma_{e_{l,n}}{}^{2}}$$

In this mode, the processing delay at the cell-edge user is reduced, and the decoder circuit power of the cell-edge user ${ҏ}_{k}^{co}$ is saved. As each cell-edge user receives their signal in this mode, the cell-edge users do not need to apply SIC, and the interference is consequently reduced.

The algorithm of the proposed mMIMO NOMA double-mode model is shown in Algorithm 1. The proposed model efficiently utilizes the battery power of the relay, and the battery power is saved at the cell-edge user.
**Algorithm 1** Massive Mimo Double-Mode ModelObtain the channel gains and assign the power coefficients $a_{l,n}\;\mathrm{and}\;b_{n}$.Compute
$$SINR_{\;R_{l}\;}=\frac{Ɓa_{l,n}{\left|h_{l}^{H}w_{l}\right|}^{2}}{\sum_{i=n+1}^{N}Ɓa_{l,i}{\left|h_{l}^{H}w_{l}\right|}^{2}+\sum_{k=1}^{n-1}a_{l,k}Ɓ{\left|h_{l}^{H}w_{l}\right|}^{2}+\sigma^{2}}$$
**For** *l* = 1: number of relay devicesObtain the range of the relay battery power $P_{l}^{{ʩ}_{s}}$, and accordingly select the operational mode.
4.(a) If the mode is ${ʩ}_{1}$, compute
$$SINR_{e_{l,n}\;}=\frac{b_{n}P_{l}{\left|g_{n}\right|}^{2}}{\sum_{\begin{array}{c} k\epsilon \Psi ,i\epsilon ƣ\\ l\neq k,i\neq n\\ g_{n}P_{l}<g_{i}P_{k} \end{array}}\sqrt{b_{i}P_{k}}{\left|g_{n}\right|}^{2}+\sum_{k=1}^{n-1}b_{k}P_{l}{\left|g_{n}\right|}^{2}+\sigma_{e_{l,n}}{}^{2}}$$If the mode is ${ʩ}_{2},$
$$SINR_{e_{l,n}\;}=\frac{b_{n}P_{l}{\left|g_{n}\right|}^{2}}{\sum_{\begin{array}{c} k\epsilon \Psi ,i\epsilon ƣ\\ l\neq k,i\neq n\\ g_{n}P_{l}<g_{i}P_{k} \end{array}}\sqrt{b_{i}P_{k}}{\left|g_{n}\right|}^{2}+\sigma_{e_{l,n}}{}^{2}}$$(b) Compute achievable data-rate and sum-rate ${Ð}_{E2E\left(n\right)}=\;log_{2}\left(1+SINR_{E2E\left(n\right)}\right)$ bps/Hz    ${Ð}_{E2E}{}^{T}=\;\sum_{i=1}^{N}{Ð}_{E2E\left(i\right)}$ bps/Hz   **End.**

## 4. Performance Analysis

The performance analysis of the proposed mMIMO NOMA double-mode model is shown in this section and compared with the performance of mMIMO NOMA.

A network system with total Ň users and a single BS, centered in a cell of 500 m radius, is considered. The users are randomly spread all over the cell. To analyze the performance, the user count Ň varies from 150 to 450 users. The total number of considered relays is L, and each relay transmits the signal to n cell-edge users at a maximum. The considered numerical parameters for the scenario are given in Table 3. A comparative analysis of ‘mMIMO NOMA’ and the proposed ‘mMIMO NOMA double-mode model’ is carried out through simulations.

**Figure 3:**
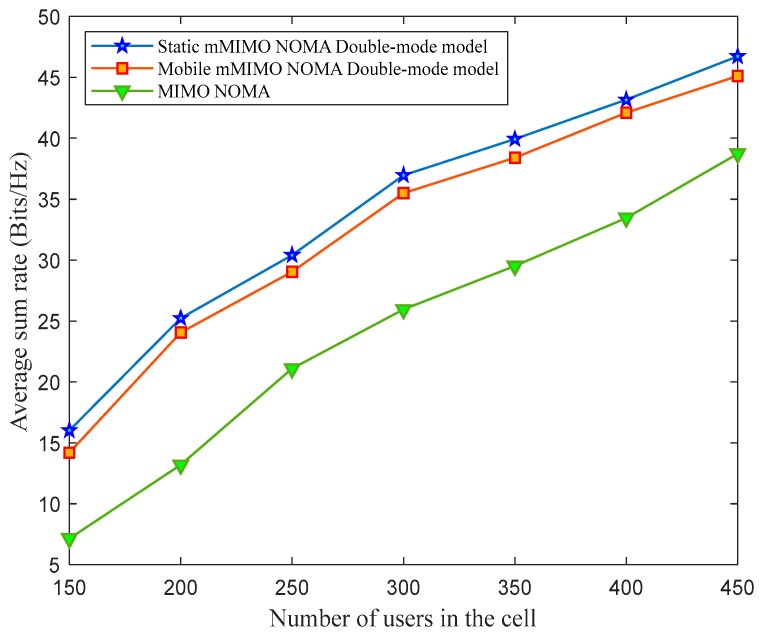
The average sum rate for different numbers of users in the cell.

Figure 3 shows the average sum rate for the considered mMIMO NOMA and the proposed mMIMO NOMA double-mode model with static users and mobile users cases. An increment in the average sum rate is noticed with the increase in the number of users in the cell. In the mMIMO NOMA double-mode model, the inter-NOMA interference $\sum_{k=1}^{n-1}b_{k}P_{l}{\left|g_{n}\right|}^{2}$ is mitigated in operational mode ${ʩ}_{2}$; therefore, $SINR_{e_{l,n}}$ increases in this mode. Consequently, the data-rate ${Ð}_{E2E\left(n\right)}$ and the sum-rate ${Ð}_{E2E}{}^{T}$ increase in this mode, which has a positive impact on the average sum-rate of the mMIMO NOMA double-mode model. Therefore, it can be seen from Figure 3 that the average sum rate of the mMIMO NOMA double-mode model is superior to the mMIMO NOMA system. When static mMIMO NOMA double-mode model and mobile mMIMO NOMA double-mode model are compared, the performance of the mobile mMIMO NOMA double-mode model is inferior to that of the static mMIMO NOMA double-mode model due to the mobility and the channel gain fluctuations. The system achieves an improved performance; however, with an increase in the number of users, the resource requirements and hardware complexity increase. As each antenna requires individual RF units for radio signal processing, an extensive increase in number of users will increase the complexity of processing. Hence, high, computationally intensive signal processing and hardware tools would be required.

**Figure 4:**
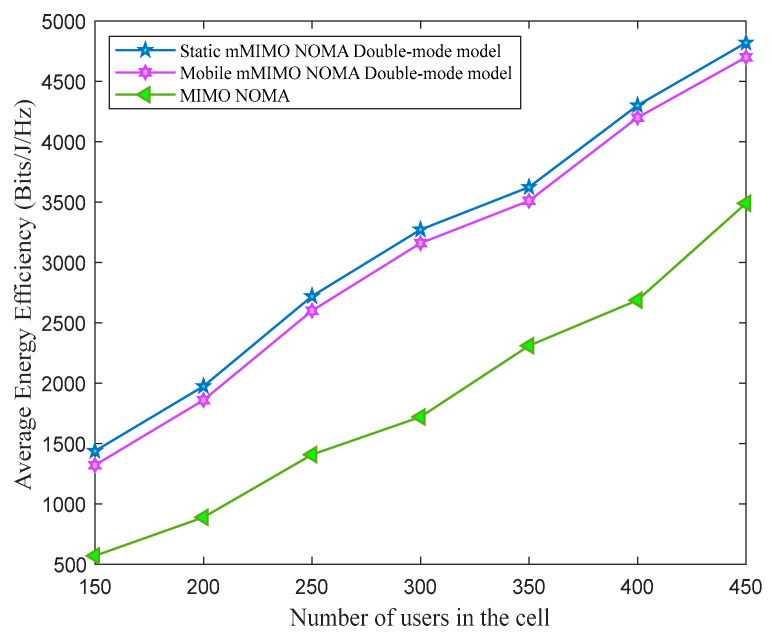
The average system’s energy efficiency for different numbers of users in the cell.

From Figure 4, it can be observed that the system’s average energy efficiency increases with the surge in the number of users in the cell. Moreover, the performance superiority of the static and mobile mMIMO NOMA double-mode model can be observed in Figure 4 in terms of average energy efficiency. According to the operation of the mMIMO NOMA double-mode model, in the operational mode ${ʩ}_{2}$, the relay uses more battery; therefore, more power is devoted to battery power than to its decoder circuit. Therefore, the proposed mMIMO NOMA double-mode model ensures that all the relays have sufficient battery levels even after decoder circuit power consumption, which is an effective way of utilizing the relay battery power. It can be seen from Figure 3 that the rates of the mMIMO NOMA mMIMO NOMA double-mode model are superior to those of the mMIMO NOMA system. Referring to (16) and (17), as energy efficiency is proportional to the rates, this positively impacts the average energy efficiency of the mMIMO NOMA double-mode model.

**Figure 5:**
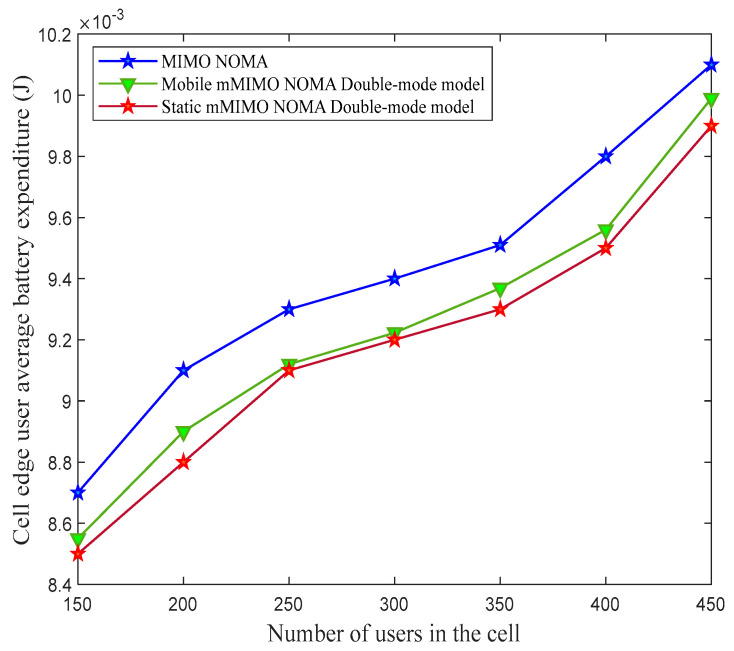
The average battery power expenditure of the cell-edge user for different numbers of users in the cell.

The average expenditure of the battery power of the cell-edge user in the proposed static and mobile mMIMO NOMA double-mode model was analyzed for different numbers of users in the cell and compared to the mMIMO NOMA system. Figure 5 shows that the overall power expenditure at the cell-edge user in the proposed mMIMO NOMA double-mode model is reduced by the reduction in the decoder circuit power in operational mode ${ʩ}_{2}$. This decreases the value of ${ҏ}_{n}$; hence, the cell-edge user’s battery power expenditure is also reduced.

## 5. Conclusions

This paper studied a cooperative mMIMO NOMA system in a single cell with a random distribution of users. A novel efficient clustering technique and mMIMO NOMA mMIMO NOMA double-mode model scheme were proposed, which choose the operational modes according to the batter power of the cooperative relay device. The proposed mMIMO NOMA mMIMO NOMA double-mode model scheme is observed to outperform the mMIMO NOMA scheme regarding average sum rate, average energy efficiency, and cell-edge users’ average energy expenditure. The proposed scheme efficiently utilizes system energy efficiency by using the relayand cell-edge user’s battery power efficiently. Furthermore, the proposed system reduces overall power expenditure for the cell-edge user by reducing the decoder circuit power in operational mode. As the mMIMO NOMA mMIMO NOMA double-mode model optimizes the system’s energy, it paves the way for a green wireless communication network. Using the simulation results, the proposed technique’s effectiveness can be validated through various performance parameters.

As a future research direction, the mMIMO NOMA double-mode model could be employed with higher-frequency bands such as mmWave and THz channels as an alternative to the RF channel to improve the system’s spectral efficiency mmWave and THz band, and offer larger bandwidths. Employing higher-frequency bands might restrict the communication distance; therefore, this is a challenge for future research.

## Figures and Tables

**Table 1:** Comparative analysis of this paper with existing works.

Ref. No.	Description of Algorithm	Optimization Method	Key Performance Indicators	Efficient Clustering	mMIMO NOMA Double-Mode Model for Power Optimization
[14]	In the power line communication system, average capacity is studied for OMA and NOMA cooperative systems	Simulation-based	Average capacity	×	×
[15]	Global energy efficiency in cooperative NOMA systems is analyzed	Bilateral optimization problem	Energy efficiency	×	×
[16]	In NOMA, a dynamic method of resource allocation for sub-channel assignment is studied	Lyapunov, Lagrangian optimization, two- side matching and linear method	Energy efficiency	×	×
[17]	A comparative analysis of OMA and NOMA in MIMO system is carried out in terms of sum-rate	Simulation-based	Sum-rate	×	×
[18]	OMA and NOMA techniques are analyzed to improve fairness in MIMO system	Simulation-based	Fairness	×	×
[19]	Non-regenerative mMIMO NOMA relay system	Simulation-based	Capacity and sum-rate	×	×
Proposed Scheme	A mMIMO NOMA double-mode model chooses its modes of operation based on the relay battery level	Simulation-based	Energy efficiency, sum-rate, and cell-edge user battery consumption	√	√

**Table 2:** Notations.

Ň	Overall cell users
N	Cell-edge users
Ä	Total antennas at the BS
Ψ	Relay users set
ƣ	Cell-edge users set
L	Total number of relay users
$R_{l}$	lth relay
$e_{n}$	nth cell-edge user
${Ɓ}_{max}$	Maximum BS power
$P_{l}$	Battery power of $R_{l}$
$x_{l}$	Transmitted signal from BS to lth relay
$y_{R_{l}}$	The signal received at $R_{l}$
$h_{l}$	Channel between BS and $R_{l}$
$g_{n}$	Channel between $R_{l}$ $\mathrm{and}\;e_{l,n}$
$SINR_{R_{l}}$	Signal-to-noise-ratio at $R_{l}$
$SINR_{e_{l,n}}$	Signal-to-interference noise-ratio at $e_{l,n}$
$\sigma^{2}$	BS-relay link AWGN variance
$\sigma_{e_{l,n}}{}^{2}$	The variance of AWGN in the $R_{l}$ to $e_{l,n}$ link
${Ϫ}_{n}$	$e_{l,n}$ amplifier drain efficiency
${Ԃ}_{l}$	$R_{l}$ amplifier drain efficiency
$EE^{T}$	Total energy efficiency
${Ð}_{E2E\left(n\right)}$	Data rate at data-rate of BS-$R_{l}$ $\mathrm{and}\;R_{l}-e_{l,n}$ end-to-end link
${Ð}_{E2E}{}^{T}$	Total sum rate
${Ϸ}_{l}$	The total energy consumed by $R_{l}$ for transmitting signal for $e_{l,n}$
${Ƥ}_{l}$	$R_{l}$ power expenditure at its circuit
${ҏ}_{n}$	Circuit power expenditure of *n*th cell-edge user
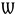 _*l*,*n*_	Power expenditure at $e_{l,n}$

**Table 3:** Numerical parameters.

Parameters	Value
Frequency of operation	2 GHz
Number of users in the cell, Ň	150 to 450
Total number of antennas at BS, Ä	128
Total time	100 s
Speed of the users	10 m/s
Radius of the cell	500 m
Distance between BS and relay users	300 to 400 m
Distance between BS and end-users	400 to 500 m
Maximum battery power of BS, ${Ɓ}_{max}$	42.7 dBm
Antenna beamwidth of the relay in the azimuth plane, ${¥}^{T}$	$90^{\circ }$
Relay and end-user receiver noise power, $\sigma^{2},\sigma_{e_{l,n}}{}^{2}$	−128.23 dBm
Range of relay battery power, $P_{l_{max}}-P_{l_{min}}$	−50 dBm to 10 dBm
Battery power of relay in mode ${ʩ}_{1}$$,\;P_{l}^{{ʩ}_{1}}$	−50 to 6.99 dBm
Battery power of relay in mode ${ʩ}_{2}$ $,\;P_{l}^{{ʩ}_{2}}$	6.99 to 10 dBm
Amplifier drain efficiency of cell-edge users ${Ϫ}_{n}$, and relay amplifier ${Ԃ}_{l}$	0.75%
Path loss between user t (km) distance apart forming direct links [24]	$148+40\;log_{10}\;(t$) dB
Path loss and shadow from the base station	$148.1+37.6\;log_{10}\;\left(t\right)$ dB
Channel gain formula, for a given path loss	$10^{-\left(Path\;Loss/10\right)}$

## Data Availability

Not applicable.
